# Is the Regular Intake of Anticoagulative Agents an Independent Risk Factor for the Severity of Traumatic Brain Injuries in Geriatric Patients? A Retrospective Analysis of 10,559 Patients from the TraumaRegister DGU^®^

**DOI:** 10.3390/brainsci10110842

**Published:** 2020-11-12

**Authors:** Nicolas Eibinger, Sascha Halvachizadeh, Barbara Hallmann, Franz Josef Seibert, Paul Puchwein, Till Berk, Rolf Lefering, Kai Sprengel, Hans Christoph Pape, Kai Oliver Jensen, The TraumaRegister DGU

**Affiliations:** 1 Division of Trauma Surgery, Department of Orthopaedics and Trauma, Medical University of Graz, 8036 Graz, Austria; nicolas.eibinger@medunigraz.at (N.E.); franz.seibert@medunigraz.at (F.J.S.); paul.puchwein@medunigraz.at (P.P.); 2 Department of Trauma, University Hospital Zurich, 8091 Zürich, Switzerland; Sascha.Halvachizadeh@usz.ch (S.H.); Till.Berk@usz.ch (T.B.); Kai.Sprengel@usz.ch (K.S.); Hans-Christoph.Pape@usz.ch (H.C.P.); 3 Department of Anaesthesiology and Intensive Care Medicine, Medical University of Graz, 8036 Graz, Austria; barbara.hallmann@klinikum-graz.at; 4 Institute for Research in Operative Medicine (IFOM), University of Witten/Herdecke, 58455 Witten, Germany; rolf.lefering@uni-wh.de; 5 Committee on Emergency Medicine, Intensive Care and Trauma Management (Sektion NIS) of the German Trauma Society (DGU), 80639 Munich, Germany; traumaregister@auc-online.de

**Keywords:** traumatic brain injury, medication, geriatric, odds, DOAC

## Abstract

The purpose of this study was to assess anticoagulant medication as an independent factor influencing the occurrence of a severe traumatic brain injury in geriatric patients. Data were collected from the TraumaRegister DGU^®^ between January 2015 and December 2018. We included patients with an age of ≥65 years with a blunt TBI; an AISHead ≥2 but no other relevant injuries. Patients were divided into five subgroups: no anticoagulant medication, anti-platelet drugs, vitamin K antagonists, direct-oral-anticoagulants, and heparinoids. Separation between moderate TBI (AISHead 2–3) and severe TBI (AISHead ≥ 4) and multivariable regression analysis were performed. The average age of 10,559 included patients was 78.8 years with a mean ISS of 16.8 points and a mortality of 22.9%. The most common cause of injury was a low fall of <3 m with 72.8%. With increasing age, the number of patients without any anticoagulant therapy decreased from 65.9% to 29.9%. The intake of coagulation medication increased mortality significantly. Severe TBI was observed in 51% of patients without medication and ranged from 61 to 67% with anticoagulant drugs. After adjusting for confounding variables, the intake of VKA or DOACs was significantly associated with an increased risk of severe TBI. The use of anticoagulant medication is an independent factor and is associated with an increased severity of TBI depending on the type of medication used.

## 1. Introduction

Traumatic brain injuries (TBIs) are one of the leading causes of death and disabilities worldwide. The incidence of TBIs in Europe is estimated to be 1000 per 100,000 people [1]. The highest incidence occurs in older adults, with a strong increase at the age of 75 and above [2]. The average hospitalization rate for TBI among geriatric patients (65 years and older) is nearly four times higher than that of younger patients [3]. Due to the demographic changes in the population, the number of TBIs will increase over the next years. Currently, the number of trauma patients older than 60 years increases by 1.6% each year [4]. The same trend can be seen for the number of people regularly treated with anticoagulative medication [5,6].

The influence of anticoagulative therapies on the outcome and severity of TBIs is a controversial topic in the literature. Some studies reported a worse outcome for all types of anticoagulative drugs, whereas, in other studies, significantly worse outcomes are reported for vitamin K antagonists and Direct oral anticoagulants (DOACs) [7].

The aim of the current study was to investigate the influence of anticoagulative drugs as an independent risk factor on the severity of TBI in geriatric patients.

## 2. Materials and Methods

The TraumaRegister DGU^®^ (TR-DGU) of the German Trauma Society (Deutsche Gesellschaft für Unfallchirurgie, DGU) was founded in 1993. The aim of the TraumaRegister DGU^®^ is to document severely injured patients using multi-pseudonymized and standardized documentation. 

Data were collected prospectively in four consecutive time phases from the time of the accident until discharge from hospital: (A) pre-hospital phase; (B) emergency room and initial surgery; (C) intensive care unit; (D) discharge. The inclusion criterion was admission to the hospital via the emergency room with subsequent ICU/ICM care or arrival at the hospital with vital signs and death before admission to the ICU.

The participating hospitals submitted their pseudonymized data into a central database via a web-based application. Scientific data analysis was approved according to a peer review procedure laid down in the publication guidelines of TraumaRegister DGU^®^.

The participating hospitals were primarily located in Germany (90%), but a number of hospitals in other countries contributed data as well (including from Austria, Belgium, China, Finland, Luxembourg, Slovenia, Switzerland, The Netherlands, and the United Arab Emirates). Participation in TraumaRegister DGU^®^ was voluntary. For hospitals associated with TraumaNetzwerk DGU^®^, the entry of at least one basic data set is obligatory for reasons of quality assurance.

The present study is in line with the publication guidelines of the TraumaRegister DGU^®^ and registered as TR-DGU project ID 2019-009. 

In the TR-DGU all injuries are coded based on the Abbreviated Injury Scale (AIS, version 2005; 2008 update) [8].

### 2.1. Study Population

Patients’ data were collected from the TraumaRegister DGU^®^. 

#### 2.1.1. Inclusion Criteria

All patients older than 65 years, who have suffered from a blunt TBI with an AIS_Head_ ≥ 2, without any relevant injury to another AIS region (AIS_other then head_ ≤ 2) were included. Additionally, patients must have been admitted to a European trauma center between January 2015 and December 2018.

#### 2.1.2. Exclusion Criteria

All patients with missing documentation of age, a penetrating or unknown trauma mechanism, and incomplete documentation of coagulation medication were excluded. Details are shown in Table 1. 

### 2.2. Definitions 

To define TBI, only AIS codes starting with the number 1 were used. According to Lefering et al., a non-relevant additional injury was defined with an AIS ≤ 2 [9]. Moderate TBI was defined with AIS codes 2 and 3 and severe TBI with an AIS code higher than 4. 

For further analysis, the patients were divided into five groups:

(1) No anticoagulant medication (NM);
(2) Anti-platelet drugs (APD);
(3) Vitamin K antagonists (VKA);
(4) Direct oral anticoagulants (DOACs);
(5) Heparinoids.

### 2.3. Statistical Analysis

Statistical analyses were performed using SPSS statistical software (SPSS Version 24, IBM Inc., Armonk, NY, USA). Data were presented as mean with standard deviation (±SD) and as percentages for categorical variables. Multivariable logistic regression analysis with a severe TBI (AIS 4/5/6) as the dependent variable was performed to estimate the impact of coagulation medication intake on the severity of the TBI (measured by the AIS). Therefore, the multivariable logistic regression model was adjusted for the potential confounders type of accident, age, gender, ASA score, and additional injuries in other body regions. Odds ratios (OR) are presented with their respective 95% confidence interval (CI). Due to multiple comparisons, formal statistical testing was avoided, and statistical significance was mentioned only in selected situations. The level of significance was set at *p* < 0.05.

## 3. Results

**Figure 1:**
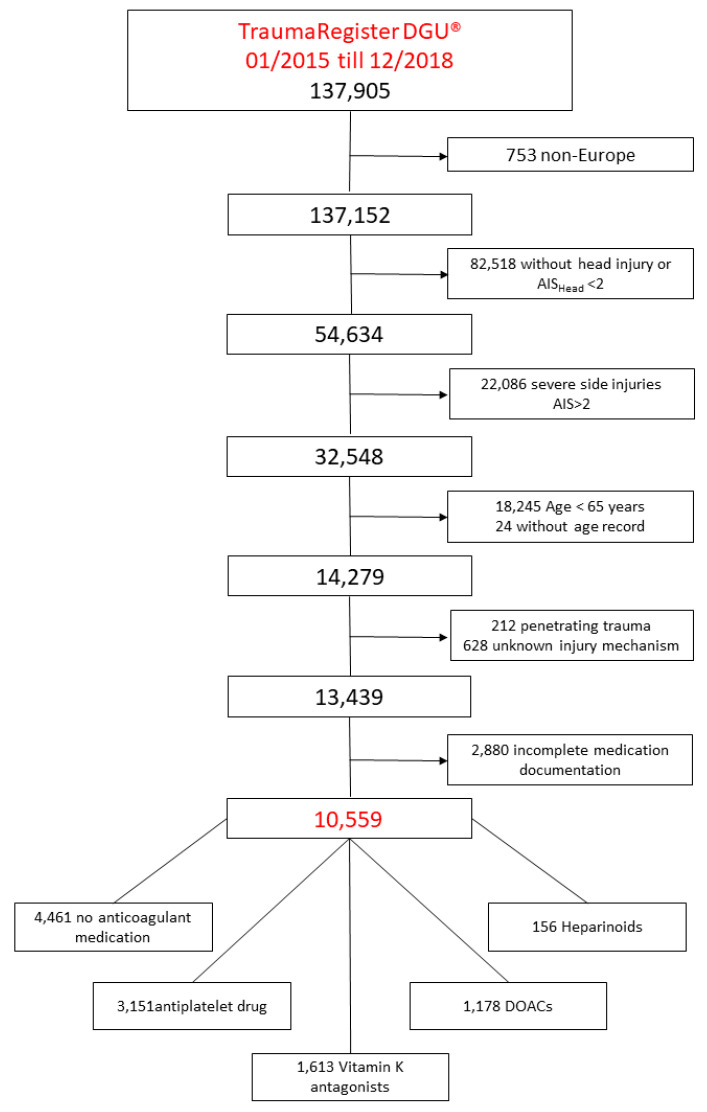
Study outline.

A total of 137,905 patients were documented in the TraumaRegister DGU^®^ between January 2015 and December 2018. Out of these patients, 10,559 fulfilled the inclusion criteria (Figure 1). The mean (SD) age of all patients was 78.8 (±7.6) years. The youngest patients were found in the NM group with an average of age 76.6 (±7.7) years. The mean ISS was 16.8 (±8.3) points. Basic data are shown in Table 2.

The most common cause of injury was a low fall from less than 3 m (72.8%), followed by bicycle accidents (9.3%), and falls from above 3 m (6.8%). Only 0.9% suffered injuries after a motorcycle accident.

**Figure 2:**
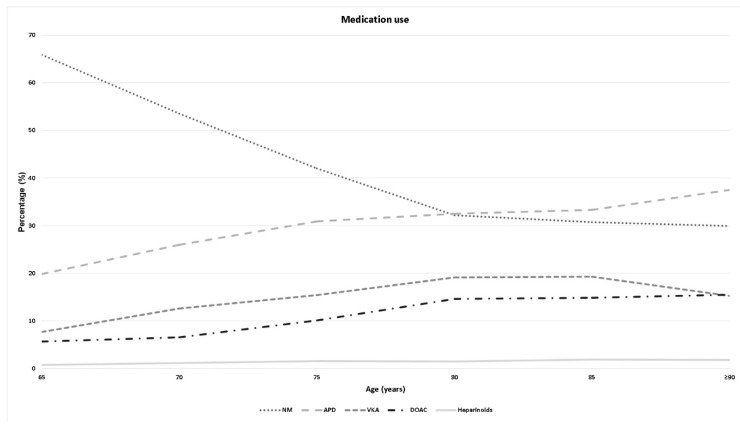
Illustration of medication use throughout different age groups. NM: no anticoagulant medication; APD: antiplatelet drugs; VKA: vitamin K antagonist; DOAC: direct oral anticoagulants.

With increasing age, the percentage of patients using antithrombotic medication increased from 34.1% at the age of 65–69 years to 70.1% in patients older than 90 years. The highest increase was observed in the APD group, where the percentage increased from 19.9 to 37.5% (Figure 2).

The overall observed mortality rate was 22.9%. Only 7.5% deceased when they endured a moderate TBI, whereas the number increased by up to 34.8% in patients that suffered from a severe TBI. Pre-injury use of any coagulation medication increased the mortality rate significantly even in moderate TBIs (Table 3).

Overall, 56.6% of the patients included in this study suffered from a severe head injury (AIS = 4+). The lowest portion of severe head injuries was observed in patients not taking any blood diluting medications (51.3%), whereas the percentage increased significantly with the intake of VKA to 66.5% (Table 4). 

The unadjusted OR for intake of anticoagulative medication was significant for all four drugs (OR from 1.23 to 1.89; Table 2). However, patients taking no drugs were significantly younger (*p* < 0.001), less severely injured (ISS, *p* < 0.001), and had a better health status regarding ASA (*p* < 0.001). After adjusting for selected confounders (type of accident, age, gender, ASA score, additional injuries in other body regions) the intake of anticoagulative medication was still significant only for VKA and DOAC: OR 1.43 and 1.24, respectively (Table 3).

The presence of any injuries besides the TBI was associated with a reduction in the number of severe TBIs. Out of all patients who suffered an isolated TBI, 67.7% were categorized as severe. This number decreased with the presence of an injury AIS_other than head_ 1 to 48.5% and AIS_other than head_ 2 to 44.1%. 

## 4. Discussion

In an ageing society, the increasing number of geriatric trauma patients will be one of the greatest challenges in the coming years [10]. Over the last few years, the number of severely injured geriatric patients has continued to increase because of an increased life expectancy and a higher level of activity and mobility. Due to comorbidities, the treatment of these patients requires immediate specialized care [11].

The purpose of this study was to assess the role of anticoagulant medication as a potential independent factor influencing the occurrence of severe traumatic brain injuries in the geriatric population.

Our main results are as follows:

1. The rate of patients taking anticoagulative medication increased in the geriatric population with increasing age.
2. The use of anticoagulant medication increased the severity of TBI in geriatric patients as an independent risk factor.
3. The type of anticoagulant medication was associated with the severity of TBI.
4. Pre-injury intake of anticoagulant medication increased the mortality rate.

As expected, the data demonstrated that the use of antithrombotic or anticoagulant medication increased with the growing age of patients [5,6,12,13,14]. In the general patient population the number of patients with any form of blood dilutive pharmacotherapy increased by up to 70.1%. The use of blood dilutive medication alters the severity of head injuries. The influence of different subtypes of medications is discussed controversially in the literature [14,15,16,17,18,19,20,21,22,23]. In this study, the number of severe TBIs was higher in each subgroup compared to the patients without any blood dilutive pharmacotherapy.

In our results, pre-injury use of any coagulation medication led to an increased mortality rate, even in moderate TBI. The influence of ADP on the mortality rate, in particular, has been discussed controversially in the literature [7,14,16,22,24]. However, based on the available data, we were unable to differentiate between APD monotherapy or dual antiplatelet therapy, which lead to an increased mortality rate according to the literature [14,16,25]. The mortality rates of the VKA and DOAC subgroups were comparable to each other and with the current literature [12,15,20,22,26,27]. We observed the highest mortality rate in the heparinoid group. We were not able to find any data in the literature about the influence of preinjury use of heparinoids on overall mortality rates from TBI.

Another finding was regarding the influence of additional injuries on TBI. Similar data were observed by Banerj et al. [28]. A possible reason for the reduction in severe TBI could be that the patients try to reduce the speed of the fall. This, for example, could have meant that the patients broke the fall with their arms, or hit the ground with a body part other than their head. 

After adjusting the OR, patients with preinjury intake of VKA and DOACs were found to have a significantly higher risk of suffering a severe TBI. This suggests that seniors who fall while under the influence of these medications sustain more severe head injuries. This leads to more complications and to higher mortality rates [14,15,19,23]. The results show that the use of DOACs does not seem to be superior to VKA in the case of TBI. Not enough data were available to assess the influence of missing reversal agents, [29].

The use of anticoagulant monitoring parameters (e.g., viscoelastic test, international normalized ratio) in TBI is becoming more important in clinical practice, and targeted coagulant therapy might be a new way to improve the outcome of TBI [30].

Certain limitations of this investigation must be acknowledged. As the present study had a retrospective character, all findings represent associations and do not claim any causality. Registry data are less valid than data obtained in prospective randomized trial setting, but the data quality in the TR-DGU is verified every three years at each participating trauma center. The comparability of groups is questioned since baseline characteristics are different. Due to the software of the TraumaRegister DGU^®^, the recording of more than one anticoagulative drug is not possible, and thus we were not able to analyze the influence of the simultaneous intake of different types of anticoagulative agents. Another limitation was that the AIS was the only way of measuring the presence of a TBI. No radiographic parameters were available at the dataset. No mild TBIs were included due the inclusion criteria of the TraumaRegister DGU^®^ and the calculation of the ISS. Still, it is unclear how many bleedings occur after moderate trauma due to preexisting anticoagulation treatments.

## 5. Conclusions

The current study showed that the preinjury intake of VKA or DOACs is an independent risk factor for the severity of TBI, and that it is associated with higher mortality rates, in geriatric trauma patients. The data also showed that the intake of these drugs rapidly increased with raising age and that most patients suffered their injuries from a low energy trauma. Due to the high percentage of low falls, it is recommended that efforts should be made regarding fall prevention, and there is the need to weigh the risk of thrombotic events against the risk of intracerebral hemorrhages. Therefore, the development of new clinical risk-prediction tools could be useful.

## Figures and Tables

**Table 1:** Detailed inclusion and exclusion criteria.

Inclusion Criteria
Age ≥ 65 years
AIS_Head_ ≥ 2
AIS_other then head_ ≤ 2
Blunt trauma mechanism
Complete anticoagulant medication documentation
Admitted to a European trauma center
**Exclusion Criteria**
Unknown age or <65 years
AIS_Head_ < 2
AIS_other then head_ ≥ 3
Penetrating or unknown trauma mechanism
Incomplete anticoagulant medication documentation
Admitted to a non-European Trauma centre

**Table 2:** Base data of the patient collective. NM: no anticoagulant medication; APD: antiplatelet drugs; VKA: vitamin K antagonist; DOAC: direct oral anticoagulants; ISS: Injury severity score; ASA: American Society of Anaesthesiologists score.

	NM	APD	VKA	DOACs	Heparinoids	Total
Number	4461	3151	1613	1178	156	10,559
Proportion (%)	42.2	29.8	15.3	11.2	1.5	100
Age (Mean ± SD)	76.6 (±7.7)	80.1 (±7.3)	80.4 (±6.8)	81.2 (±7.0)	80.8 (±7.4)	78.8 (±7.6)
ISS (Mean ± SD)	16.0 (±8.0)	16.7 (±8.2)	18.6 (±8.6)	17.9 (±8.5)	18.0 (±8.5)	16.9 (±8.3)
AISHead	3.5	3.7	3.9	3.8	3.9	3.7
ASA 3/4 (%)						50.1
Male (%)						54.9

**Table 3:** Mortality rates and 95% confidence interval for moderate and severe TBI according to preinjury anticoagulation medication intake. No significant differences between the subgroups in relation to age or injury severity where found.

	Moderate TBI	95% Confidence Interval	Severe TBI	95% Confidence Interval	Total	95% Confidence Interval
NM	5.3		27.9		16.9	
APD	8.2	6.8–9.5	34.8	32.6–37.0	23.2	21.7–24.7
VKA	10.7	8.1–12.6	43.9	40.9–46.9	32.7	30.4–35.0
DOAC	10.1	7.3–12.3	41.7	38.2–45.2	30.1	27.5–32.7
Heparinoids	23.0	12.4–31.5	45.2	35.2–55.2	36.5	28.9–44.1
Total	7.5		34.8		22.9	

**Table 4:** Multivariate Logistic regression analysis with severe TBI (AIS 4/5/6) as a dependent variable. OR for different types of anticoagulation therapy (reference group is no medication), without adjustment and after adjusting for age, gender, type of accident, and concomitant injury.

	OR for Severe TBI	95% Confidence Interval
Unadjusted Odds Ratios		
APD	1.23	1.12–1.34
VKA	1.89	1.67–2.21
DOAC	1.63	1.43–1.86
Heparinoids	1.48	1.07–2.05
Adjusted Odds Ratios		
APD	1.03	0.93–1.15
VKA	1.43	1.25–1.63
DOAC	1.24	1.07–1.43
Heparinoids	1.11	0.78–1.57
